# Factors Associated with Fertility Intention among Chinese Married Youth during the COVID-19 Pandemic

**DOI:** 10.3390/bs13020184

**Published:** 2023-02-17

**Authors:** Ruicheng Peng, Wei Mou, Peng Xu

**Affiliations:** Department of Sociology, School of Philosophy, Zhongnan University of Economics and Law, Wuhan 430073, China

**Keywords:** married youth, fertility intention, COVID-19, socioeconomic status, parenting perception

## Abstract

Using the childbearing survey data from Hubei Province in March 2022, this article empirically analyzed the status quo of fertility intention and its influencing factors among Chinese married youth during the COVID-19 pandemic. In our analysis, fertility intention was operationalized as the ideal number of children and short-term fertility plan. Statistical results showed that the average ideal number of children stood at 1.652, which was lower than the population replacement level, whilst only 16.4% of married youth had a short-term fertility plan. By utilizing a binary logit regression model and the sheaf coefficient technique, we found that COVID-19-induced factors (i.e., change in the marital relationship during the epidemic, delayed pregnancy preparation due to vaccination) had a more stable effect on fertility intention, especially on short-term fertility planning. Parenting perception characteristics exerted a great impact on the ideal number of children but a relatively small impact on short-term fertility planning. Meanwhile, married youth with stable jobs and a high family income did not necessarily show stronger fertility intentions than those with fewer socioeconomic resources. In addition, the findings also reveal that the relative importance of fertility-influencing factors could vary at different fertile stages, which have valuable implications for population policy in Chinese contexts.

## 1. Introduction

Driven by the accelerating industrialization process and the national fertility policy, China has undergone profound changes in demographic structure and the characteristics of fertility since the 21st century [1]. According to the Seventh National Census data, the total fertility rate of Chinese women of childbearing age fell to 1.3 by the end of 2020, indicating that China has entered a period of extremely low fertility levels [2]. Currently, the fertility anxiety mentality is gradually permeating Chinese society, especially among individuals aged under 35 [3]. In order to alleviate the severe dilemma of low fertility, the Chinese government has successively promulgated a “selective two-child policy (i.e., the husband or wife who is an only child can have two kids)”, the “universal two-child policy”, and the “three-child policy”. However, the pro-birth policies have failed to bring about the expected population growth, and some young people even suffer from “childbearing phobia” [4]. Meanwhile, the problem of the low fertility rate has been increasingly aggravated under the COVID-19 pandemic. Although the spread of COVID-19 has recently been effectively controlled, the epidemic per se led to a series of unintended social consequences. Specifically, the stay-at-home quarantine policy during the pandemic inevitably increased family economic pressures and enhanced work–family conflicts, which could further affect the fertility intentions of reproductive women. Moreover, mass vaccinations also raised concerns about the side effects of the COVID-19 vaccine in pregnant women. In fact, the nationwide economic downturn, accompanied by the deteriorating employment environment, undoubtedly added more uncertainties to household fertility decisions at the present stage [5,6,7,8]. There has been evidence presented that the size of the Chinese population born in 2020 decreased by 18% compared with that in the previous year [9]. Thus, it could be inferred that the novel coronavirus outbreak might serve as one kind of social inducement affecting married persons’ fertility intentions.

In the existing literature, social scientists often co-opt the Theory of Planned Behavior (TPB) to analyze the possible constraints of fertility intentions. As per Ajzen’s theoretical framework, fertility behaviors should be considered the outcome of “life choices” associated with childbearing decision-making processes, which are consequentially restrained by certain “life chances” (i.e., personal and contextual conditions) [10,11]. For instance, some studies inspired by the TPB pointed out that fertility intentions were related to individual socioeconomic status, including household income, human capital (i.e., education/health), and employment quality [12,13,14,15]. Other scholars emphasized that subjective factors such as parenting perceptions were also attributed to the heterogeneity of fertility intentions [16]. Nevertheless, prior research has usually focused on the general population of childbearing age, rather than young adults, who are key intervention objects of fertility policy. In addition, the effect of the COVID-19 pandemic on fertility intention was understated, especially in non-western contexts such as Chinese society. In fact, Chen et al. revealed that the COVID-19 pandemic reduced the fertility intention of Chinese women, largely ascribed to the increased economic pressure and the conflict between work and childbearing [5]. However, existing studies still lacked comparisons of the relative importance of various kinds of fertility intention determinants, which ought to be conducive to optimizing fertility promotion policy. Furthermore, from the perspective of policy practice, exploring the constraints of fertility intention is an important basis for the formulation of population development policies, while the association between relevant factors and fertility intentions could vary according to socio-cultural circumstances in different countries [17]. The previous comparative analysis provided valuable evidence that marital fertility in China was not only lower than in the West but that Chinese families’ childbearing decisions (e.g., the number and sex composition of the offspring) seemed to be more influenced by deliberate control [18]. At present, under the background of the three-child policy starting from May 2021, the fertility structure of Chinese families gradually became more diversified, which would further highlight the importance of distinguishing the fertility intentions between childless, one-child, and two-child families. In spite of this, insufficient attention has been paid to the heterogeneous effects of fertility-influencing factors at different steps of Chinese family reproduction [19]. In this study, using the data from a recent large-scale childbearing survey in Hubei Province, we intend to address the above-mentioned oversights in previous research by exploring the status quo of fertility intention and its influencing factors among married Chinese youth during the COVID-19 pandemic. In particular, based on descriptive statistical analysis, we will further quantitatively identify the relative effect sizes of different factors associated with fertility intentions. Admittedly, in view of medical sociology, the field of fertility practice is not reducible to the issue of individual childbearing decisions, whilst any “choice” of fertility behaviors ought to be affected by both individual agency and social relational factors [20]. In this sense, our attempt to analyze the relative importance of fertility-influencing factors may help to provide illuminating policy implications for Chinese population development.

## 2. Literature Review and Research Questions

Fertility intention is a multi-dimensional concept involving individuals’ expectations of the number, gender, and timing of children [21]. Although some scholars underlined the differences between fertility intention and subsequent reproductive behavior [22,23], most empirical investigations conducted in both developed and developing countries have demonstrated that fertility intention is an effective indicator of future fertility decision-making [24,25,26,27]. Specifically, fertility intention generally includes two interrelated aspects, i.e., ideal family size and an actual fertility plan. The former refers to a person’s desire to reproduce regardless of potential obstacles, which reflects the need for children and does not contain any commitment to action. The latter points to such behavioral tendencies that individuals choose to have children after having a fertility desire, with more emphasis on commitment, planning, and operability. Compared with reproductive desire, the actual fertility plan is more easily restricted by environmental changes and individual characteristics [28]. In line with the theoretical framework of TPB and relevant literature, the current study tends to categorize the personal and contextual determinants of fertility intention into three main dimensions, i.e., objective socioeconomic status, subjective parenting perceptions, and COVID-19-induced factors. In the following part, we will briefly elaborate on the correlations between these factors and fertility intention and then put forward specific research questions.

Individual socioeconomic status is frequently mentioned in previous research that aims to predict fertility intentions and behaviors. Relevant studies generally harness proxy variables such as occupation, income, and education to measure the level of socioeconomic status. Indeed, under the context of sluggish economic development, international and domestic researchers have paid close attention to the effect of employment status on fertility intention, positing that economic precariousness (e.g., unstable employment, low salaries) would give rise to the postponement of parenthood [29,30,31,32]. Moreover, Vignoli et al. found that the effect of job uncertainty on fertility intention would further be indirectly mediated by subjective well-being, especially for parents and older individuals [33]. Similar evidence from East Asian countries also suggests that turbulent labor market conditions and associated work–family conflicts were also important predictors of fertility intention [34,35]. As per Beck’s theory of risk society, wealth is usually accumulated within the higher social class, while risk aggregates primarily in the lower social class. In other words, the ability to cope with social risk will be unequally distributed across the economic hierarchy [36]. Beck’s viewpoints suggest that those who lack economic resources tend to defer parenthood decisions due to insecure economic basis. Additionally, education level is also recognized as an important socioeconomic marker affecting individuals’ performance in labor markets [37], but its effects on fertility seem to be relatively complicated. Most previous analyses pointed out that individuals with higher education had increased opportunities to participate in career life, which would lead to later ages of childbearing in order to maximize the return on human capital investment [38,39,40]. Even so, there was inconsistent evidence that the effect of female education on fertility should be generally small and possibly heterogeneous [41]. Moreover, Martín-García and Baizán emphasized that the types of education, i.e., learning about the care of individuals or involving social skills or relational capacities, might be as important as the education level in determining the timing of first birth [42].

Apart from objective socioeconomic status, a couple’s subjective perceptions of parenting also play a significant role in the process of fertility decision-making. Under the context of intensive social competition, a growing number of Chinese parents feel anxious about whether or not they could give their children enough material resources to achieve higher prestige. Such a negative psychological state may lead to a decrease in the likelihood of having a child. For example, Xu and Pak once treated child-raising and fertility decisions as a Tullock contest model. They found that competitive pressure to allocate parenting resources resulted in the overaccumulation of human capital and low fertility [43]. Similarly, Zerle-Elsäßer and Gniewosz argued that the insufficiency of familial resources (e.g., quality of co-parenting) was negatively linked to mothers’ subjective well-being, which might be relevant for further fertility intentions [44]. The theory of quality–quantity tradeoff provides theoretical support for explaining the influence of subjective perceptions of parenting on fertility intention. As proposed by Becker and Lewis [45], parents tend to invest more resources in parenting to improve their children’s quality (e.g., educational achievements) and maximize family utility. When family parenting resources are limited, the pursuit of children’s quality will lead to reduced demand for the number of children. Within current Chinese society, the three-child policy primarily concentrates on encouraging couples to have more children, along with a remarkable rise in the opportunity costs of childbearing and childrearing. Thus, it could be inferred that the possible gaps between pro-birth policy and subjective perceptions of parenting are likely to bring about the heterogeneity of fertility attitudes and behaviors [46].

In addition, the worldwide outbreak of the COVID-19 crisis has already induced a vast array of unexpected consequences on everyday life, contributing to the non-negligible change in fertility traits [47]. In light of this, we ought not to ignore the potential impact of COVID-19-related factors on fertility. To be specific, empirical evidence from western countries suggested that some adults of a fertile age lowered their fertility intention partly due to the increase in economic uncertainty and unemployment levels [48,49]. Compared with western countries, the Chinese government adopted a stricter “dynamic zero clearance” policy in response to high infection risks, which inevitably exerted a tremendous impact on the order of social life (e.g., mandated physical distancing, postponed re-opening of schools and childcare facilities, etc.). Zhou and Guo found that over half of Chinese respondents who planned to give birth intended to change their childbearing plans in cases of epidemics [50]. On the one hand, the evident decline in fertility intention partly pertained to stronger work–family conflict and worse marital well-being during the COVID-19 pandemic [51,52]. On the other hand, coronavirus vaccination served as an important means of epidemic prevention and control. However, residents’ potential concerns about vaccine safety for pregnant women might further negatively affect the fertility intentions of those of childbearing age [53].

In short, there has been a large body of published literature that theoretically or empirically discussed the issue of fertility intention and its social determinants. Although these illuminating studies have recognized the effects of different factors on fertility, more in-depth knowledge about the relative importance of various factors (e.g., objective socioeconomic status, subjective parenting perceptions, and COVID-19-induced factors) is still missing in the existing literature. Moreover, there is obvious heterogeneity in childbearing decisions at different fertile stages. For instance, based on a recent survey of 1026 couples from Shanghai, Zhu et al. pointed out that the proportions of those who planned to rear a second/third child were approximately 16% and 9%, respectively [21]. Therefore, it is necessary to distinguish the mechanism of determinants associated with fertility intention at different fertile stages. In light of the above analysis, the current study primarily attempts to identify factors related to fertility intention during the pandemic, striving to address the following specific questions:

(1) Do socioeconomic status, parenting perceptions, and COVID-19-induced factors have a significant influence on the fertility intentions of Chinese married youth? Which type of factors matters more?

(2) Are the effects of various factors on fertility intentions consistent across different fertile stages?

## 3. Materials and Methods

### 3.1. Data Source

This study was based on the Childbearing Survey in Hubei Province, which was jointly organized by the Population and Health Research Center of Zhongnan University of Economics and Law and the Yichang Municipal Health Commission. This survey was designed to investigate residents’ fertility intentions and the influencing factors under the three-child policy starting from May 2021, as well as to provide theoretical and empirical support for the construction of a “fertility-friendly society” in China. The survey subjects of childbearing age (20–45) were randomly selected in Yichang, Hubei Province. The survey scope covered the entire Yichang area, including 5 districts (Yiling District, Xiling District, Wujiagang District, Dianjun District, and Xiaoting District), 3 county-level cities (Yidu City, Zhijiang City, and Dangyang City), 3 counties (Yuan’an County, Xingshan County, and Zigui County), 2 autonomous counties (Changyang Tujia Autonomous County and Wufeng Tujia Autonomous County), and 1 high-tech zone (Yichang High-Tech Zone). A total of 32,248 samples of fertile age were collected online in March 2022. For the present study, married youth (below 35 years old) in their first marriage were selected from the database. After eliminating the data missing for key variables, 13,794 valid samples were finally obtained.

### 3.2. Measurement

The dependent variable was the fertility intention of married youth, which included two dimensions, i.e., the ideal number of children and short-term fertility plan. As for the ideal number of children, there was a question entitled “how many children would you like to have in an ideal situation”, with possible answers being “0, 1, 2, 3, or more than 3”. In order to highlight the differences in fertility intention tendency, we recoded this item into such a binary variable as “want 2 kids or more = 1, want 1 kid or none = 0”. As for short-term fertility plans, the survey asked the following questions: (1) Do you plan to have a child this year? (answered by childless respondents); and (2) do you plan to have another child this year? (answered by those who had at least one child). In fact, such two questions provided childbearing decision information about fertility timing as well the quantum. Based on the above two questions, we set up three dummy variables: “first child plan of childless family”, “second child plan of one-child family”, and “third child plan of two-child family”, with the coding “have fertility plan = 1, no short-term fertility plan = 0”, respectively.

Regarding the operationalization of socioeconomic status, in this study, objective socioeconomic status was related to the following three variables: (1) Education level (years of schooling), including a master’s degree or above = 19, 4-year university degree = 16, 3-year college degree = 15, senior high/technical school = 12, junior high school = 9, primary school = 6, uneducated = 0; (2) employment status, including stable job, unstable job, and no job; and (3) household economic status, involving the objective annual income (an ordinal variable ranging from 1 to 8) and subjective household economic status (an ordinal variable ranging from 1 to 5). We used the principal component analysis to combine these two ordinal variables into a common factor, and then recoded the common factor into a continuous variable (ranging from 0–100) by means of the min–max transformation method.

Regarding the operationalization of subjective parenting perceptions, we took into account two variables designed to identify the effect of young couples’ parenting perceptions on fertility intention. The first is educational expectations for children. There was one relevant question in the questionnaire: Which level of education do you expect your children to achieve in the future? There were three options for answering this question, namely, a master’s degree or above, a 4-year university degree, a 3-year college degree, or below. The other variable was the perception of multi-child family advantages. We used the following question: What do you think about raising multiple children compared with raising only one child? The answer options were recoded as two dummy variables, i.e., “multi-child family is better for children’s development” and “multi-child family has no obvious advantage”.

Regarding the operationalization of COVID-19-induced factors, and consistent with a previous literature review, we utilized two questions in the questionnaire to measure COVID-19-induced factors. The first question pertained to changes in the marital relationship during the COVID-19 pandemic, with the options being “more intimate”, “no change”, and “more distant”. The second relevant question was as follows: How long do you think it would be good to get pregnant after vaccination? This question reflected the delayed effect of pregnancy preparation caused by vaccination. There were four possible answers, i.e., “more than 12 months”, “6 months to 12 months”, “3 to 6 months”, and “less than three months”.

In the statistical analysis, we also introduced the following control variables: Gender (male = 1, female = 0); age group (20 to 25 years old, 26 to 30 years old, and 31 to 35 years old); household registration (agricultural registration = 1, non-agricultural registration = 0); ethnic group (Han = 1, minority = 0); and life satisfaction (satisfied = 1, unsatisfied =0). Additionally, there was evidence that the childbearing expectation was closely associated with existing fertility experience and peer group relational networks [18,54], so we further controlled for the gender composition of the child (only have a girl(s), only have a boy(s), have both a boy and a girl, no child), the time span since their last birth (in years), and peer influence (most friends have kids = 1, only a few friends have kids = 0).

### 3.3. Statistical Methods

The statistical analysis was conducted in three steps. Firstly, we used descriptive methods to demonstrate the distribution characteristics of the current fertility structure in married Chinese youth. Next, based on binary logit regression models, we intended to examine the social determinants of fertility intention (i.e., the ideal number of children and short-term fertility plan). In order to measure the relative contribution of different types of factors, all independent variables were simultaneously added to the regression model. Specifically, the statistical model is as follows:

(1) $$\mathrm{logit}\left(\mathrm{Pr}\right)=\beta_{0}+\beta_{m}x_{m}+\beta_{n}x_{n}+\beta_{p}x_{p}+\beta_{q}x_{q}$$

In Equation (1), Pr represents the probability of fertility intention. β_0_ is the intercept term of the model. $x_{m}$, $x_{n}$, and $x_{p}$ represent the variables socioeconomic status, subjective parenting perceptions, and COVID-19-induced factors, respectively, while $x_{q}$ is a group of control variables.

Then, based on the above-mentioned logistic regression model, we further used the sheaf coefficient technique proposed by Heise [55]. Supposing that there are three latent variables indicating socioeconomic status ($\eta_{1}$), subjective parenting perceptions ($\eta_{2}$), and COVID-19-induced factors ($\eta_{3}$), their corresponding relationships with the three groups of independent variables are as follows:

(2) $$\eta_{1}=c_{1}+z_{m}x_{m}$$

(3) $$\eta_{2}=c_{2}+z_{n}x_{n}$$

(4) $$\eta_{3}=c_{3}+z_{p}x_{p}$$

Thus, Equation (1) can be rewritten into the following alternative form:

(5) $$\mathrm{logit}\left(\mathrm{Pr}\right)=\beta_{0}+\lambda_{1}\eta_{1}+\lambda_{2}\eta_{2}+\lambda_{3}\eta_{3}+\beta_{q}x_{q}$$

Equation (5) is the equivalent form of Equation (1) using the iterative method. $z_{m}$, $z_{n}$, and $z_{p}\;$are three sets of post-estimated parameters. The three latent variables ($\eta_{1},\;\eta_{2},\;\eta_{3}$) are standardized, and their standard deviations equal 1. By doing this, the effect sizes of the three latent variables on fertility intention become comparable within the same equation. In the following analyses, we harnessed STATA software to perform the aforementioned regression procedure and “sheafcoef” command.

## 4. Research Results

### 4.1. Descriptive Statistics of Fertility Structure and Fertility Intention in Married Youth

Table 1 shows the basic characteristics of the current fertility structure in married youth. In general, the proportion of single-child families was the highest at 65.7%, while that of two-child families and childless families accounted for 19% and 15.3%, respectively. In terms of the cohort difference, the 20- to 25-year-old age group occupied the highest percentage of childless families and the lowest percentage of two-child families. Regarding household registration, compared with non-agricultural samples, the proportion of childless married youth with agricultural household registration was slightly higher. In addition, the proportion of childless and one-child families of minority youth was slightly higher than that of Han youth, whilst the proportion of two-child families presented the opposite tendency. With an increase in years of schooling, the proportion of childless and one-child families showed a growth trend, but the proportion of families with two children gradually declined. Furthermore, compared with families with a lower economic status, families with a better economic status showed a higher proportion of being childless and a lower proportion of second children, which indicated that a family’s economic situation might not necessarily be the decisive factor for fertility intention. It is also worth mentioning that although China’s three-child policy had been implemented for more than half a year before this survey, there were no samples of married youth who reared a third child, reflecting the grim reality that the implementation of the three-child policy has not met expectations.

Next, based on the two dimensions of fertility intention (ideal number of children and short-term fertility plan), we further explored the possible inducers of low fertility intention. As shown in Table 2, the average ideal number of children in married youth was 1.652, while the percentage of those who had a short-term fertility plan only accounted for 16.4%. In terms of demographics, the fertility intention indicators were higher in male and minority samples than in their counterparts. As for COVID-19-induced factors, respondents who had a worse marital relationship and had concerns about the side effects of vaccination tended to have lower fertility intentions. In addition, young people with higher socioeconomic status (a bachelor’s degree or above, a stable job, and higher family income) were more likely to have a short-term fertility plan. Finally, in terms of parenting perceptions, young people who believed that having more children should be more beneficial for children’s development presented higher fertility intention, but there seemed to be a nonlinear relationship between the expectation of children’s education and fertility intention. In summary, the results of preliminary bivariate analyses indicated that heterogeneous characteristics existed in married youth’s fertility intention. Thus, it is necessary to further apply the logistic regression method to carry out in-depth quantitative analyses regarding the determinative patterns of fertility intention.

### 4.2. Results of Logistic Regression Analysis

As mentioned above, this study was primarily concerned with the effects of socioeconomic status, parenting perceptions, and COVID-19-induced factors on fertility intention. Table 3 displays the estimation results based on the binary logit regression models. Among them, the dependent variable in model 1 was the ideal number of children (want 1 child or none = 0), while models 2 to 4 investigated the short-term fertility plan of childless families, one-child families, and two-child families, respectively (no short-term fertility plan = 0).

Firstly, we examined the association between socioeconomic status and fertility intention. With other variables controlled for, each additional year of schooling decreased the likelihood of wanting more kids by approximately 4.8% (OR = 0.952, *p* < 0.001). Meanwhile, education level also exerted a significant impact on the fertility plans of childless families; their probability of giving birth to another in the short term would be 9.5% lower for each additional year of schooling (OR = 0.905, *p* < 0.001). However, for families with one or two kids already, respondents’ education level did not affect their fertility plan. Moreover, household economic status was partly associated with fertility intention: Every additional unit of household economic score would increase the chance of wanting at least two kids by 3.5% (OR = 1.035, *p* < 0.01); for one-child families, every additional unit of household economic score would enhance the chance of having a second child by 9.4% (OR = 1.094, *p* < 0.001); for childless and two-child families, household economic status seemed to be non-influential upon short-term fertility plans. Moreover, the effects of occupation on fertility intention were relatively weak: Young respondents with stable jobs (managers or professional technicians in state-owned enterprises and institutions) did not show evidently higher fertility intentions than those with no job. Such results indicated that when other factors are controlled, socioeconomic status might not necessarily be a reliable and strong predictor of fertility intention. Thus, the low fertility intentions of married youth cannot simply be attributed to the lack of affordability of child-rearing.

The results of logistic regression models also showed differentiated effects of subjective parenting perception on fertility intention. Those who held the view that having more children is beneficial for children’s development presented a 199.5% higher chance to want at least two kids (OR = 2.995, *p* < 0.001). Compared with the samples who only expected their children to earn a 3-year college degree or below, the odds of having stronger fertility desire among those who expected their children to earn a master’s degree/bachelor’s degree increased by 21.8% (OR = 1.218, *p* < 0.05)/22.2% (OR = 1.222, *p* < 0.05). In terms of fertility plans, compared with the reference group, those who believed “having more children is more beneficial to children’s development” presented a 35.4% higher chance of having a second child (OR = 1.354, *p* < 0.01), while those who expected their children to obtain a master’s degree demonstrated a 61.5% (OR = 1.615, *p* < 0.05) higher chance of having a first child for childless families. In addition, the influence of parenting perception on fertility intentions of two-child families seemed to be very weak.

Lastly, we further analyzed the impact of COVID-19-induced factors on fertility intention. Since the outbreak of COVID-19 in early 2020, working-from-home has become a common lifestyle and increased the frequency of face-to-face indoor interactions. The data in Table 3 show that married youth who reported more distant marital relationships were 26.8% less likely to want more than two kids during the pandemic (OR = 0.732, *p* < 0.01); at the same time, the probability of having a short-term fertility plan for childless families and one-child families also decreased by 76.8% (OR = 0.232, *p* < 0.01) and 33.4% (OR = 0.666, *p* < 0.1), respectively. Regarding two-child families, a worse marital relationship during the pandemic did not have a statistically significant effect on short-term fertility plans. Additionally, vaccination is a necessary measure to prevent and control the epidemic. However, because young people have different levels of concern about the side effects of vaccines, vaccination per se may bring about a delay in pregnancy preparation. To be specific, there was an inverted U-shaped relationship between the length of pregnancy preparation after vaccination and the ideal number of children, with the probability of wanting at least two kids significantly higher in the group of “waiting 3 months to 1 year”. Moreover, the longer the preparation interval of pregnancy (e.g., more than half a year), the lower the likelihood of having a short-term fertility plan for all three types of families (*p* < 0.01). As a result, the potential consequences of vaccination on pregnancy were still controversial among young people, which indeed restricted their fertility intention to some extent.

### 4.3. Relative Importance of Different Factors on Fertility Intention

Although the above analyses confirmed that socioeconomic status, parenting perception, and COVID-19-induced factors exerted heterogeneous effects on the fertility intention of married youth, it was still unclear which type of factors had a greater effect and deserves more attention from policy makers. In Table 4, the sheaf coefficient technique was introduced on the basis of logistic regression models to further estimate the relative importance of different factors as latent variables. As can be seen from the statistical results in Table 4, the ideal number of children was more influenced by their subjective parenting perceptions, with an effect size 2.497 times (0.467/0.187) and 3.592 times (0.467/0.13) that of COVID-19-induced factors and socioeconomic status, respectively. However, in terms of short-term fertility plans, COVID-19-induced factors seemed to play a greater role and show a stable and significant effect. This means that effectively addressing the negative impact of COVID-19 on people’s everyday life should become an important policy direction to promote the childbearing decisions of married youth.

**Figure 1:**
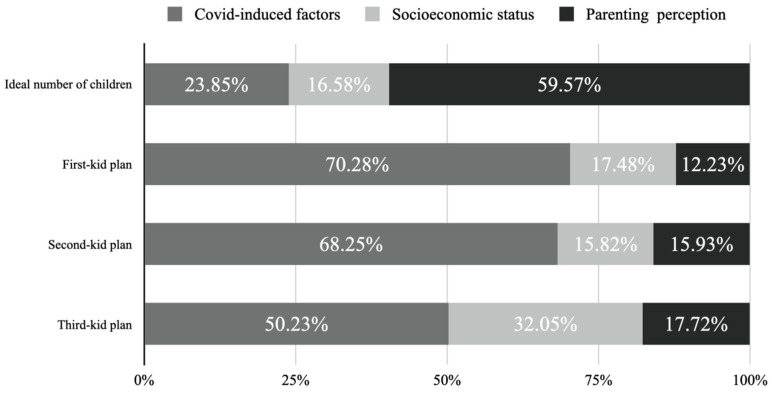
Relative contributions of three latent variables to fertility intentions.

In addition, since the sheaf coefficients are values obtained after standardized processing, the effect sizes of latent variables can thus be compared within the same model by limiting the sum of the effects of each latent variable to 1. As shown in Figure 1, COVID-19-induced factors should be very important, because they explained approximately 70% of the total variation in fertility plans caused by three latent variables amongst childless families/one-child families. In other words, COVID-19-induced variables primarily affected the timing of births but not necessarily the ultimate number of ideal births. Meanwhile, subjective parenting perception had more of an impact on the ideal number of children, while socioeconomic status showed a greater effect on the short-term fertility plans of two-child families. In general, the results indicate that fertility intention is not a single concept but rather involves a complex fertility decision-making process. Therefore, for families at different fertile stages, it is necessary to adopt differentiated fertility promotion policies according to the effect sizes of relevant factors.

## 5. Conclusions and Policy Implications

Based on a childbearing survey in Hubei Province, China, this study pointed out that one-child families still accounted for the largest part of the current family structure in Chinese married youth. In terms of their fertility intention, the average ideal number of children stood at 1.652, which was lower than the population replacement level, whilst only 16.4% of married youth had a short-term fertility plan. Thus, it can be seen that China’s long-lasting family-planning policy has exerted a strong squeezing effect on the fertility intentions of contemporary youth. In view of this, by empirically analyzing the status quo of fertility intention and its influencing factors, this article would help us better interpret the social determinants of fertility intention and promote our understanding of the low-fertility problem in China.

### 5.1. Conclusions

In the current study, we utilized a binary logit regression model and sheaf coefficient method to investigate the effects of socioeconomic status, parenting perceptions, and COVID-19-induced factors on fertility intention, and drew the following main conclusions. Firstly, COVID-19-induced factors (i.e., changes in the marital relationship under the epidemic and delayed pregnancy preparation due to vaccination) had a more stable effect on fertility intentions, especially on short-term fertility plans. Secondly, parenting perception characteristics exerted a large impact on the ideal number of children but a relatively small impact on the short-term fertility plan, indicating that actual fertility decisions were less likely to be restricted by subjective factors. Thirdly, married youth with stable jobs and high family incomes did not necessarily show stronger fertility intentions than those with fewer socioeconomic resources. Fourthly, the effects of different factors on the ideal number of children and short-term fertility plan seemed to be heterogeneous, which underlined the fact that fertility intention is related to the complex fertility decision-making process. Thus, it is inappropriate to simply use a single indicator (e.g., the ideal number of children) as its proxy variable.

### 5.2. Policy Implications

In view of these empirical findings, we intend to put forward the following policy suggestions. First of all, relevant authorities should attach great importance to the improvement of marital relationships during the COVID-19 pandemic. Professional counseling channels can be provided for couples who experience an emotional crisis so as to guide married youth to actively cope with the negative emotions caused by the epidemic. Then, it is necessary to pay attention to young people’s concerns about the side effects of the COVID-19 vaccine. The mainstream media need to report authoritative guidance on the vaccine’s potential influence on pregnant women based on experimental results, aiming to reduce the negative impact of information uncertainty on young people’s fertility intentions. Furthermore, in light of the heterogeneous effects of various factors on fertility intention, it is necessary to shift the focus of the pro-birth policy from economic assistance to providing comprehensive child-rearing services, aiming to alleviate youth’s anxiety about childbirth and construct a childbearing-friendly society. Finally, different interventions should be implemented for families at different fertile stages so as to effectively promote the actual effects of fertility policies.

### 5.3. Limitations and Prospects

There are still some limitations to this study. First, our statistical analyses were based on a cross-sectional dataset, so the causal relationships between various factors and fertility intention cannot be clearly inferred. Therefore, a longitudinal design is needed to reconfirm our findings’ robustness. Second, this childbearing survey did not strictly adhere to the random sampling approach, so the representativeness of our research findings is insufficient to some extent; Moreover, the number of samples needs to be increased in order to avoid sample selection bias. Third, due to the limitation of questionnaire data, the measurements of some variables (e.g., socioeconomic status, prior fertility experience) are not rigorous, which may affect the precision of the research results. Fourth, since fertility behavior is, in essence, “relational”, it is also required to relationally penetrate and explain how interdependent networks (e.g., peer groups) within the social field of fertility constrain the patterns of fertility practice among married youth.

## Figures and Tables

**Table 1:** Distribution Characteristics of Fertility Structure in Married Youth.

Variables	Coding	Childless Family	One-Child Family	Two-Child Family	Chi-Square Test
Age	20 to 25	42.5%	51.4%	6.1%	*p* < 0.001
	26 to 30	34.2%	56.5%	9.3%	
	31 to 35	7.6%	69.4%	23.0%	
Household registration	Agricultural	17.9%	65.7%	16.5%	*p* < 0.001
	Non-agricultural	14.2%	65.6%	20.1%	
Ethnic group	Han	14.9%	65.6%	19.5%	*p* < 0.01
	Minority	16.9%	66.1%	17.0%	
Education level	Junior high school or below	3.5%	57.4%	39.0%	*p* < 0.001
	Senior high/technical school	6.7%	68.3%	25.0%	
	3-year college	16.4%	67.5%	16.1%	
	4-year university	23.5%	64.7%	11.8%	
	Master degree or above	33.4%	55.5%	11.1%	
Household economic status	Upper	17.8%	64.3%	17.9%	*p* < 0.001
	Upper-middle	16.1%	65.8%	18.1%	
	Middle	16.6%	66.4%	17.0%	
	Lower-middle	15.8%	65.3%	18.9%	
	Lower	10.6%	66.2%	23.2%	
	N = 13,794	15.3%	65.7%	19.0%	

**Table 2:** Distribution Characteristics of Ideal Number of Children and Short-term Fertility Plan in Married Youth.

Variables	Coding	Mean of Ideal Number of Children	Have Fertility Plan	F Test/ Chi-Square Test	Sample Size
Gender	Male	1.732	23.6%	*p* < 0.001 *p* < 0.001	1895
	Female	1.639	15.3%		11,899
Age	20 to 25	1.555	29.5%	*p* < 0.001 *p* < 0.001	346
	26 to 30	1.565	28.6%		3537
	31 to 35	1.686	11.6%		9911
Household registration	Agricultural	1.671	16.0%	*p* < 0.001 *p* < 0.05	9724
	Non-agricultural	1.607	17.5%		4070
Ethnic group	Han	1.640	16.4%	*p* = 0.062 *p* = 0.685	11,193
	Minority	1.663	16.7%		2601
Change of marital relationship under the epidemic	More distant	1.544	7.5%	*p* < 0.001 *p* < 0.001	704
	No change	1.642	15.2%		11,009
	More intimate	1.741	25.7%		2081
Delayed effect of pregnancy	More than 12 months	1.612	6.7%	*p* < 0.001 *p* < 0.001	5232
	6 to 12 months	1.680	16.9%		4781
	3 to 6 months	1.692	33.3%		1985
	Less than 3 months	1.648	24.8%		1796
Education level	Junior high school or below	1.775	9.2%	*p* < 0.001 *p* < 0.001	1184
	Senior high/technical school	1.693	10.6%		3971
	3-year college	1.647	17.2%		3688
	4-year university	1.591	21.8%		4625
	Master degree or above	1.626	28.5%		326
Employment status	Stable job	1.615	21.4%	*p* < 0.001 *p* < 0.001	5471
	Unstable job	1.700	12.8%		2428
	No job	1.666	13.3%		5895
Household economic status	Upper	1.667	19.8%	*p* < 0.05 *p* < 0.001	2415
	Upper-middle	1.677	18.3%		2870
	Middle	1.634	17.1%		2905
	Lower-middle	1.646	15.3%		2796
	Lower	1.637	12.0%		2808
Expectation of children’s education	3-year college degree or below	1.601	17.9%	*p* < 0.001 *p* < 0.01	931
	4-year university degree	1.660	16.1%		5156
	Master degree or above	1.643	16.4%		7707
Perception of multi-child family advantage	Multi-child family is better for children’s development	1.859	17.4%	*p* < 0.001 *p* = 0.076	3182
	Multi-child family has no obvious advantage	1.590	16.1%		10,612
All samples		1.652	16.4%		13,794

**Table 3:** Predicting the Odds Ratio of Fertility Intention among Chinese Married Youth Based on Logistic Regression Analysis.

Variables	Model 1: Ideal Number of Children	Model 2: Childless Families	Model 3: One-Child Families	Model 4: Two-Child Families
**Socioeconomic Status**				
Education level	0.952 *** (0.010)	0.905 *** (0.034)	0.968 (0.024)	0.930 (0.088)
Household economic status	1.035 ** (0.016)	1.004 (0.004)	1.094 *** (0.037)	1.004 (0.014)
Employment status (no job = 0)				
Stable job	0.944 (0.048)	0.884 (0.120)	0.855 (0.098)	1.098 (0.561)
Unstable job	1.035 (0.057)	0.631 *** (0.112)	0.879 (0.106)	0.291 (0.226)
**Parenting Perception**				
Perception of multi-child family advantage (multi-child family has no obvious advantage = 0)	2.995 *** (0.155)	0.968 (0.125)	1.354 *** (0.131)	1.698 (0.707)
Expectation of children’s education (3-year college degree or below = 0)				
Master degree or above	1.218 ** (0.095)	1.615 ** (0.321)	0.787 (0.138)	0.724 (0.483)
4-year university degree	1.222 ** (0.096)	1.313 (0.262)	0.861 (0.151)	0.964 (0.648)
**COVID-induced Factors**				
Change of marital relationship under the epidemic (more intimate = 0)				
More distant	0.732 *** (0.073)	0.232 *** (0.080)	0.666 * (0.159)	0.470 (0.625)
No change	0.816 *** (0.046)	0.596 *** (0.082)	0.617 *** (0.070)	0.456 * (0.201)
Delayed effect of pregnancy (wait less than 3 months = 0)				
Wait more than a year	0.837 *** (0.051)	0.201 *** (0.034)	0.253 *** (0.035)	0.118 *** (0.061)
Wait six months to a year	1.165 ** (0.072)	0.565 *** (0.089)	0.569 *** (0.072)	0.262 *** (0.127)
Wait three months to six months	1.284 *** (0.094)	1.799 *** (0.343)	1.192 (0.164)	0.3 99(0.237)
**Control Variables**				
Gender (female = 0)	1.311 *** (0.074)	1.131 (0.155)	1.573 *** (0.192)	2.299 * (1.151)
Age (20 to 25 = 0)				
26 to 30	1.143 (0.139)	1.510 * (0.308)	1.860 (0.750)	0.819 (0.709)
31 to 35	1.459 *** (0.173)	2.583 *** (0.565)	1.637 (0.658)	0.246 (0.218)
Household registration (non-agricultural = 0)	1.149 *** (0.051)	1.178 (0.137)	1.217 * (0.132)	2.037 (1.074)
Ethnic group (minority = 0)	0.944 (0.046)	1.486 *** (0.189)	0.990 (0.111)	1.205 (0.700)
Life satisfaction (unsatisfied = 0)	1.183 *** (0.048)	1.518 *** (0.173)	1.532 *** (0.153)	2.869 ** (1.393)
Peer influence (only a few friends have kids = 0)	2.385 *** (0.178)	1.310 (0.308)	2.227 *** (0.274)	1.282 (0.532)
Gender composition of child (only have girl(s) = 0)				
Only have boy(s)	1.048 (0.047)		0.897 (0.079)	0.830 (0.413)
Have both boy and girl	5.375 *** (0.586)			0.461 * (0.195)
No child	0.746 *** (0.039)			
Time span since last birth			1.020 (0.013)	0.800 (0.112)
Log pseudolikelihood	−8170.149	−1106.421	−1969.273	−131.178
Pseudo R^2^	0.088	0.134	0.080	0.180
Sample size	13794	2111	9056	2627

Note: Odds ratio (OR) and robust standard errors of regression coefficients are reported in this table; * indicates *p* < 0.1; ** indicates *p* < 0.05; *** indicates *p* < 0.01.

**Table 4:** Relative Effects of Different Factors on Fertility Intention Based on Sheaf Coefficients Model.

	Ideal Number of Children	Childless Families	One-Child Families	Two-Child Families
**Socioeconomic Status** (Education Level, occupation, household income)	0.130 *** (0.021)	0.203 *** (0.056)	0.140 *** (0.044)	0.490 (0.299)
**Parenting Perception** (Perception of multi-child family advantage, expectation of children’s education)	0.467 *** (0.022)	0.142 *** (0.053)	0.141 *** (0.041)	0.271 (0.194)
**COVID-induced Factors** (Change of marital relationship under the epidemic, delayed pregnancy preparation due to vaccination)	0.187 *** (0.020)	0.816 *** (0.059)	0.604 *** (0.048)	0.768 *** (0.185)
**Control Variables**	√	√	√	√

Note: Sheaf coefficients are reported in this table (numbers in brackets are standard errors); *** indicates *p* < 0.01.

## Data Availability

The datasets generated for this study will not be made publicly available as the authors do not have permission to share the data.
